# Tracing the Path from Obesity to Diabetes: How S-Allyl Cysteine Shapes Metabolic Health

**DOI:** 10.3390/nu17213394

**Published:** 2025-10-29

**Authors:** Federica Geddo, Susanna Antoniotti, Giulia Querio, Maria Pia Gallo

**Affiliations:** 1 Department of Life Sciences and Systems Biology, University of Turin, Via Accademia Albertina 13, 10123 Turin, Italy; federica.geddo@unito.it (F.G.); susanna.antoniotti@unito.it (S.A.); 2 Department of Clinical and Biological Sciences, University of Turin, Regione Gonzole 10, 10043 Orbassano, Italy; giulia.querio@unito.it

**Keywords:** metabolic syndrome, insulin resistance, type 2 diabetes, endothelial dysfunction, S-allyl cysteine, hydrogen sulphide, reactive oxygen species, hypertension

## Abstract

**Background:** Metabolic Syndrome (MetS) is a multifactorial condition characterized by insulin resistance, dyslipidemia, hypertension, and abdominal obesity, which collectively increase the risk of type 2 diabetes mellitus and cardiovascular diseases. Lifestyle modification represents the first-line strategy in its management, whereas pharmacological interventions are complex and typically require long-term polypharmacotherapy. In this context, natural bioactive compounds with pleiotropic effects are gaining increasing attention. Among these, S-allyl cysteine (SAC), the major sulfur-containing compound derived from black garlic, has been identified as a promising candidate due to its well-documented antioxidant and anti-inflammatory properties. **Methods:** This narrative review examines the pathophysiological mechanisms underlying MetS and summarizes current evidence on the protective role of SAC against key pathological features of this condition, including oxidative stress, inflammation, glucose and lipid dysmetabolism, endothelial dysfunction, and gut microbiota alterations. **Results:** Preclinical studies indicate that SAC counteracts lipid accumulation, insulin resistance, endothelial dysfunction, and gut dysbiosis through multiple mechanisms, including hydrogen sulfide release, reactive oxygen species scavenging, inhibition of advanced glycation end products, and modulation of metabolic pathways. **Conclusions:** SAC emerges as a promising nutraceutical for the prevention and management of MetS and its complications. This underscores the broader relevance of nutraceuticals as promising tools in mitigating metabolic dysfunctions and reducing the burden of cardiometabolic diseases.

## 1. Introduction

Metabolic Syndrome (MetS) is a cluster of metabolic disorders, including, among others, insulin resistance, dyslipidemia, and hypertension, that significantly increase the risk of developing type II diabetes (T2DM) and cardiovascular disease (CVDs) [1,2]. The first step in the management of MetS is lifestyle modification, with the adoption of a healthy diet and regular physical activity. Since there is not a single pharmacological intervention, current pharmacotherapy requires prolonged use of multiple medications to manage its various clinical components of MetS [3].

For this reason, there has been a growing interest in recent years in bioactive natural compounds that may prevent the onset of CVDs, due to the protective effects on endothelial dysfunction (ED) and hypertension, along with the antioxidant, anti-inflammatory, and glucose metabolism-modulating properties.

S-allyl cysteine (SAC) is the most abundant sulfur-containing compound derived from black garlic, which is used as a dietary supplement and in traditional medicine. SAC antioxidant and anti-inflammatory activities are well known in the literature, as well as several biological properties [4,5].

The present review aims to analyze the pathophysiological mechanisms underlying the onset of MetS and to explore the management of MetS, with a particular focus on the new pyramid of Mediterranean Diet. Furthermore, the work examines the origin of SAC from garlic maturation, safety, and bioavailability, and the current evidence on its protective effects on several clinical outcomes associated with MetS. Finally, the potential involvement of the gut microbiota and the effects of SAC on its function are discussed.

## 2. Definition and Epidemiology

Metabolic Syndrome is a pathological condition characterized by the concurrent presence of several metabolic dysregulations, including insulin resistance, hyperinsulinemia, dyslipidemia, abdominal obesity, and hypertension [1]. It is clinically associated not only with an increased risk of developing T2DM, but also with accelerated atherosclerosis driven by chronic inflammation and vascular endothelial dysfunction, conferring a significantly increased cardiovascular risk [2].

Although MetS has been defined slightly differently over the years by various organizations, its diagnosis typically relies on six key clinical indicators: waist circumference, fasting glucose levels, triglyceride levels, high-density lipoprotein (HDL) levels, cholesterol levels, and blood pressure [1,6].

The incidence of MetS is closely related to that of obesity and T2DM, together reaching a pandemic dimension [6]. Indeed, the prevalence of obesity increased worldwide, as the body mass index (BMI) rose from 21.7 kg/m^2^ in 1975 to 24.2 kg/m^2^ in 2014 in men, and from 22.1 kg/m^2^ to 24.4 kg/m^2^ in women [7,8]. The same trend is confirmed by the increase in diabetes globally, from 4.3% in 1980 to 9.0% in 2014 in men, and from 5.0 to 7.9% in women, resulting in an increase in adults with diabetes worldwide from 108 to 422 million [8,9,10].

Taken together, it is not surprising that the prevalence is also on the rise. As evinced by Noubiap et al., it varies depending on the diagnostic criteria applied [11]. In general, the increase is higher in urban than rural areas and is generally higher in women than in men. Moreover, the prevalence is significantly higher in the Eastern Mediterranean Region (34.6%), the Americas (33.4%), and Europe (31.5%) compared to Africa (23.1%) [8,11].

## 3. Pathophysiological Mechanisms

Despite the fact that pathological mechanisms underlying the development of MetS are not yet fully elucidated, visceral obesity and the resulting inflammation, along with the insulin resistance, hypertension, and endothelial dysfunction, appear to be key elements in the onset of MetS and its progression to T2DM and CVDs [2].

### 3.1. Visceral Obesity and Inflammation

Although BMI is widely used as a simple index to monitor the obesity prevalence at the population level, it has been proven to be an insufficient biomarker of the extreme variation in visceral fat distribution between individuals [12,13]. Indeed, an excess in intra-abdominal adipose tissue, also known as visceral adipose tissue (VAT), is associated with increased visceral fat accumulation, adipocyte dysfunction, chronic inflammation, and adipokine dysregulation, contributing to a higher risk of developing metabolic disorders [12].

Adipose tissue is an endocrine organ that expresses and secretes various adipokines, both pro-inflammatory, such as leptin, tumor necrosis factor-α (TNF-α), and interleukin-6 (IL-6), and others with anti-inflammatory effects, such as adiponectin [14]. Leptin is a peptide hormone primarily secreted by adipocytes that, in physiological conditions, suppresses food intake and controls glucose homeostasis, by activating a specific receptor, with isoforms expressed in the central nervous system, adipose tissue, endothelium, and pancreatic β cells [14,15,16].

Interestingly, significantly elevated plasma leptin levels have been observed in obese individuals, leading to the concept of ‘leptin resistance’, a condition characterized by a decreased tissue-responsiveness to leptin [1,17]. Moreover, leptin plays a pro-inflammatory role by stimulating the T-helper cell proliferation and the production of cytokines, such as IL-6 [17].

In contrast, adiponectin is an adipocyte-derived hormone with anti-diabetic, anti-atherogenic, and anti-inflammatory activity [18]. Plasma adiponectin levels are significantly lower in obese individuals compared to healthy subjects, particularly those with visceral fat accumulation. In addition, low adiponectin levels are also associated with an increased incidence of T2DM and CVDs [17,18], while high adiponectin level are associated with a lower risk of developing MetS [19].

As mentioned before, obesity induces an infiltration of macrophages in adipose tissue, which secrete pro-inflammatory cytokines such as IL-1β and TNF-α, that activate the c-Jun *N*-terminal kinase (JNK) and nuclear factor-kappa B (NF-kB) pathways [20]. This inflammatory condition is a typical pattern present in MetS, which takes the name of ‘low-grade chronic inflammation’, and could be considered the link between obesity, T2DM, and MetS [20,21].

### 3.2. Insulin Resistance

Insulin is an endocrine peptide hormone secreted by the pancreatic β-cells, which plays a key role in maintaining glucose and lipid homeostasis. The intracellular insulin actions are mediated by the binding to the insulin receptor (INSR) on the plasma membrane of target cells, leading to the phosphorylation of downstream proteins, including the insulin receptor substrate (IRS), phosphoinositide 3-kinase (PI3K), and AKT isoforms. This signaling cascade initiates the cellular insulin response in target tissue, ultimately promoting glucose uptake and anabolic metabolic pathways [22,23,24].

Indeed, under physiological conditions, insulin promotes glucose uptake and storage by increasing the expression and translocation of the GLUT4 glucose transporter and stimulating glycogen synthesis in skeletal muscle. In the liver, insulin enhances glycogen synthesis while inhibiting gluconeogenesis, and, finally, in the adipose tissue, it suppresses lipolysis and promotes glucose uptake and lipogenesis [22].

When this regulatory system becomes disrupted, it leads to insulin resistance (IR), a condition characterized by a reduced responsiveness of insulin-target tissue to physiological levels of insulin, resulting in the inhibition of cellular glucose uptake, gluconeogenesis, lipolysis, and glycogenolysis. As a result, insulin secretion is enhanced, leading to hyperinsulinemia [22,23,25,26]. The most reported consequence of IR is the β-cells destruction and the onset of T2DM and Metabolic Dysfunction-Associated Steatotic Liver Disease (MASLD) [25,27].

Although the mechanisms underlying the onset of IR are not yet fully elucidated, several studies agree that one of the risk factors involved in its pathogenesis is lipid oversupply, resulting from high-fat, high-calorie diets or excessive adipose tissue lipolysis. Indeed, once the adipose tissue expandability is exceeded, lipid intermediates, such as diacylglycerols (DAGs), ceramides, and free fatty acids (FFAs), also accumulate in non-adipose tissue, leading to lipid-induced toxicity (lipotoxicity) and contributing to the development of IR in liver and muscle [24,25].

One of the earliest theories proposed to explain the mechanisms of lipid-induced insulin resistance in skeletal muscle was postulated by Randle and colleagues in the 1960s. According to the so-called ‘Randle cycle’, an acute increase in fatty acid oxidation increases mitochondrial acetyl-CoA levels, inactivating pyruvate dehydrogenase (PHD) activity, which increases intracellular citrate levels and inhibits phosphofructokinase 1 (PFK-1), a key glycolytic enzyme. This leads to an accumulation of intramyocellular glucose-6-phosphate (G6P), resulting in decreased hexokinase activity, increased glucose accumulation, and reduced glucose uptake in skeletal muscle [28].

Moreover, chronically elevated FFA levels result in increased intracellular DAG accumulation, which impairs insulin signaling through the activation of specific protein kinase C (PKC) isoforms (PKC-θ in skeletal muscle and PKC-ε in liver). This activation inhibits insulin receptor signaling by reducing insulin-stimulated tyrosine phosphorylation of IRS-1 and IRS-2, PI3K activation, and downstream insulin signaling, contributing to the development of IR in both muscle and liver tissues [23,24,29].

Hyperinsulinemic euglycemic clamp (HEC) is considered the gold standard for assessing insulin resistance. However, due to its complexity, invasiveness, and cost, this test is primarily used in small-scale research and not for large population studies. A simpler and widely validated method that can be used for clinical and epidemiological purposes is the homeostasis model assessment of insulin resistance (HOMA-IR), which estimates IR based on the relationship between fasting plasma glucose and insulin levels, providing an indirect but practical measure of insulin sensitivity [30]. Results from several clinical studies show that high values of HOMA-IR are associated with an increased risk of developing both T2DM and hypertension [31].

### 3.3. Hypertension: Focus on Endothelial Dysfunction and the Role of Gasotransmitters

As already described, MetS is closely associated with inflammatory phenomena, and, in particular, vascular inflammation is very common in its pathophysiological manifestation [32]. The metabolic abnormalities that characterize MetS are strictly related to one of its earliest and most apparent vascular manifestations: endothelial dysfunction (ED) [33]. In fact, in MetS, endothelial cells lose some of their normal functions, such as regulating permeability and vascular tone, and modulating coagulation and inflammatory processes [33,34].

Reactive oxygen species (ROS), which under physiological conditions help in maintaining cellular homeostasis and proper vascular function, can increase in MetS due to a lack of the buffering capacity of cellular antioxidant systems, leading to oxidative stress. At the vascular level, the increase in ROS causes what is known as endothelial activation, which, in addition to increased secretion of pro-inflammatory cytokines, leads to a reduction in the main gasotransmitters involved in regulating the vascular tone, nitric oxide (NO) and hydrogen sulfide (H_2_S) [32].

A decrease in NO results in a reduced endothelial-mediated vasodilatory effect and ED. In an oxidative environment, such as that associated with MetS, a reduction in NO bioavailability can be caused by its reaction with the superoxide anions to form peroxynitrite, resulting in further cell damage. Alternatively, ROS have a direct inhibitory effect on endothelial nitric oxide synthase (eNOS), which inevitably causes a reduction in NO release by endothelial cells [32,35,36].

To characterize ED in patients with MetS, several animal models have been studied, showing the typical traits of this multifactorial condition, distinguished by visceral adiposity, hyperinsulinemia, high blood pressure, and endothelial dysfunction characterized by high ROS and uncoupled eNOS, leading to reduced NO availability [37,38,39].

Remarkably, other studies emphasize the significant impact of reduced H_2_S levels in MetS. H_2_S is another gasotransmitter that plays a crucial role in regulating intracellular redox signaling and vascular tone. Indeed, a downregulation of the expression of cystathionine-γ lyase (CSE) and cystathionine-β synthase (CBS), the main enzymes involved in H_2_S synthesis, and a reduced vasodilatory effect of an H_2_S donor, NaHS, are closely related to eNOS impairment and therefore to ED associated with MetS [38,40].

Collectively, these studies show that MetS involves an alteration in the interplay between H_2_S and NO, and therefore in the regulation of vascular tone. Targeting the synthesis pathways of these key gasotransmitters to restore their balance could be an effective strategy for preventing and treating ED associated with MetS.

**Figure 1:**
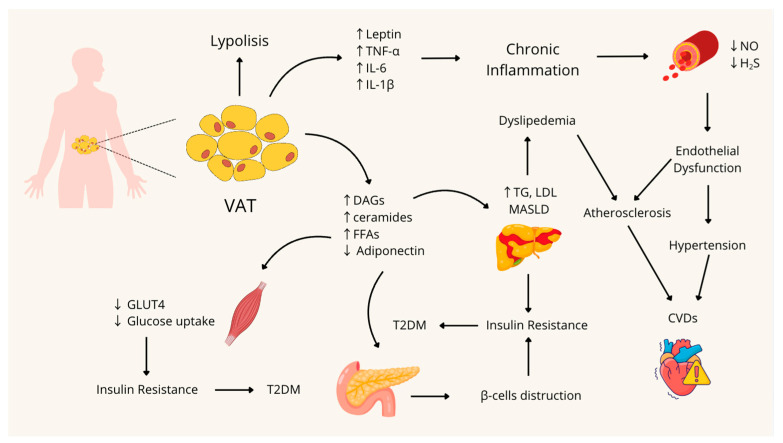
Pathophysiological mechanisms involved in the development and onset of Metabolic Syndrome. VAT: Visceral Adipose Tissue; TNF-α: Tumor Necrosis Factor-alpha; IL-6: Interleukin 6; IL-1β: Interleukin 1β; NO: nitric oxide; H_2_S: Hydrogen Sulfide; DAGs: Diacylglycerols; FFAs: Free Fatty Acids; TG: Triglycerides; LDL: Low-Density Lipoprotein; MASLD: Metabolic dysfunction-associated steatotic liver disease; CVDs: Cardiovascular Diseases; GLUT4: Glucose Transporter type 4; T2DM: Type 2 Diabetes Mellitus; ↑: Increase; ↓: Decrease. Figure created with Canva.com.

A schematic summary of the pathophysiological mechanisms involved in the onset of MetS is shown in Figure 1.

### 3.4. Role of Gut Microbiota in the Development of Metabolic Syndrome

The gut microbiota (GM) is composed of trillions of microorganisms residing in the gastrointestinal tract, including commensal, symbiotic, or pathogenic species. Its composition is highly variable and can be influenced by several factors, such as age, sex, diet, body weight, and the use of medications [41]. The GM plays different crucial roles, above all, maintaining the integrity of the intestinal barrier and preventing the colonization of pathogenic microorganisms. Moreover, since humans lack the enzymes required to digest certain complex dietary components, such as dietary fibers, the GM contributes to the digestion and adsorption of carbohydrates, by fermenting these substrates into the short-chain fatty acids (SCFAs), primarily acetate, propionate and butyrate, which provide approximately the 10% of the body’s daily caloric requirements [41,42,43]. Besides SFCAs, GM produces a wide range of metabolites with several bioactivities. Some can be produced directly from dietary components, such as tryptophan metabolites (e.g., indoles) and choline-derived metabolites (e.g., Trimethylamine *N*-oxide, TMAO), while others originate from the host and are subsequently modified by the GM, such as the secondary bile acids. Finally, some metabolites can be produced de novo by specific microbial species, such as polysaccharide A, which exerts important immunomodulatory functions [43,44]. Over the past decades, increasing evidence has highlighted a connection between GM alterations and the onset of MetS. Dietary habits, such as excessive food intake and a diet rich in sugars and fats, together with reduced physical activity, strongly influence the composition of GM. In particular, gut dysbiosis represents a key feature commonly observed in patients with obesity, T2DM, and MetS [45]. The PREDICT 1 study assessed the gut microbiome of 1,098 volunteers and associated gut microbiome structure with habitual diet and blood cardiometabolic markers. The results show that both microbial diversity and composition are influenced not only by diet but also by the food quality. Moreover, visceral adiposity, rather than BMI, shows a stronger association with GM composition. In terms of cardiometabolic health, significant correlations were observed between GM and inflammatory biomarkers (GlycA) and Very Low-Density Lipoprotein (VLDL) particle size, both of which are linked to increased risk for MetS, CVDs, and T2DM. Similarly, glycemic indicators such as insulin and C-peptide were also associated with human gut microbiome composition, together with derived predictors of insulin sensitivity and hepatic steatosis [45].

### 3.5. The Management of Metabolic Syndrome, from Diet to Pharmacotherapy

The management of MetS relies on an integrated approach that primarily involves lifestyle modifications, including dietary interventions and regular physical activity, complemented with pharmacological therapies when necessary.

The so-called Western diet (WD), originating in the industrialized Western countries, is characterized by a high consumption of high-calorie but low-nutrient food, including soft drinks, red meat, fast foods, and highly processed food, which are enriched in added sugars, salt, and saturated fats [46]. Numerous studies have linked the WD not only with metabolic disorders such as hyperinsulinemia, insulin resistance, dyslipidemia, low-grade inflammation, but also with gut dysbiosis and even mental health illness [46,47,48].

Among the dietary patterns proposed for the prevention of MetS, the Mediterranean Diet (MedDiet) is the most extensively studied and well-documented [49,50,51,52,53]. Recently, a new version of the traditional MedDiet pyramid has been produced by the Italian Society of Human Nutrition (SINU) and introduces the concept of biodiversity and sustainability. The base of the pyramid, which represents foods to be consumed on a daily basis, includes fruits and vegetables and high-quality extra-virgin oil is, which is nowadays recognized as protective against CVDs. Wholegrain cereals (pasta, rice, and bread), nuts, seeds, and dairy products (in particular milk and yogurt) must be consumed daily. The central section regards foods that are sources of plant or animal protein and have to be consumed weekly. Legumes and fish, together with fresh dairy products, are the major recommended protein sources. The upper section of the central part of the pyramid includes hard cheeses, meats, eggs, and potatoes. The top of the pyramid is occupied by products that have to be consumed occasionally: red and processed meat, together with products containing large amounts of added sugars. In the new pyramid, alcohol is represented on the left-hand side, indicating a maximum moderation in its use. Moreover, in the area below the pyramid, several key recommendations are depicted and include hydration, physical activity, social meals, seasonal foods, herbs and spices, and sustainable, biodiversity-conscious choices [54].

Adherence to this type of diet has been associated with a reduction in the prevalence and incidence of MetS. Very recently, Bruna-Mejias and colleagues evaluated through a meta-analysis the metabolic markers and clinical indicators in 2013 participants diagnosed with MetS and adhering to MedDiet or other therapeutic modalities. The main findings show that MedDiet offers significant benefits in various metabolic markers in comparison to standard diets, such as a significant reduction in BMI, waist circumference, triglycerides, blood glucose, total cholesterol, and insulin levels [55].

The second key intervention in the management of MetS, and included in the new MedDiet pyramid, is regular physical activity, which plays a crucial role in reducing weight and blood pressure, and improving insulin sensitivity and lipid disorders [56]. In a study with 4746 adults aged 55 to 75 years with metabolic syndrome and overweight or obesity, without prior cardiovascular disease or diabetes, the authors evaluated whether an energy-reduced MedDiet plus physical activity reduces diabetes incidence compared with a standard MedDiet. The results show that over a median 6-year follow-up, diabetes incidence was 31% relatively lower in the intervention group compared with the control group [57], highlighting that diet alone is not sufficient for the management of MetS, but rather a comprehensive, multifactorial approach is required.

When lifestyle modifications alone are insufficient, pharmacological therapy is necessary, especially to manage hypertension, dyslipidemia, and insulin resistance. Major treatments include Statins, which reduce hepatic cholesterol synthesis and the amount of Low-Density Lipoprotein (LDL) levels in the body, and Metformin, the most widely used insulin-sensitizing agent, which improves insulin resistance by inhibiting hepatic glucose production and enhancing glucose uptake in adipose tissue and skeletal muscle [58].

Since there is no single therapy strategy for the management of MetS, and long-term pharmacological interventions are often associated with side effects, growing attention has been directed toward bioactive plant-derived compounds as complementary or alternative strategies. In this scenario, S-allyl cysteine, a major organosulfur compound derived from black garlic, has emerged as a promising candidate due to its antioxidant, anti-inflammatory, anti-hypertensive, and insulin-sensitizing properties.

## 4. S-Allyl Cysteine

### 4.1. Origin and Derivation from Garlic Maturation, Safety, and Bioavailability

S-allyl cysteine (SAC) is a water-soluble sulfur-containing amino acid and represents the main bioactive organosulfur compound in black garlic, also commonly referred to as aged garlic. Black garlic is produced through a carefully regulated aging process conducted under controlled thermal conditions (40–60 °C) and relative humidity (60–90%), without the addition of exogenous substances. This process aims to reduce the content of molecules responsible for the pungent and unpleasant flavor of fresh garlic, while simultaneously enriching the levels of health-promoting bioactive compounds [59,60].

SAC in black garlic is formed via both enzymatic and non-enzymatic pathways. The enzymatic pathway involves the hydrolysis of endogenous γ-glutamyl-S-allyl-L-cysteine (GSAC) by the γ-glutamyl transferase enzyme (γ-GTP). This reaction is triggered during aging or when garlic cloves are chopped, as vacuolar γ-GTP becomes cytosolic and can interact with GSAC. The non-enzymatic pathway, first highlighted by Chen et al. [61], involves the thermal degradation (pyrolysis) of alliin, the major sulfur-containing amino acid of fresh garlic, and explains the increase in SAC concentration during heat processing of garlic at temperatures above 40 °C, when γ-GTP becomes inactivated.

Notably, within the food manufacturing sector, multiple pre-aging interventions—such as high-pressure processing (HPP), cryogenic freezing, and ultrasound treatment—have been systematically investigated as alternatives to conventional thermal processing, with the objective of preserving γ-GTP activity and thereby enhancing the accumulation of SAC in aged garlic. Among these strategies, HPP has consistently demonstrated superior efficacy in retaining γ-GTP enzymatic functionality and promoting SAC biosynthesis during subsequent aging stages [60].

The bioavailability and pharmacokinetics of SAC following oral administration have been extensively investigated in mice, rats, dogs, and humans, and were comprehensively summarized in a recent review [62], which highlighted the molecule’s high bioavailability and remarkable plasma stability.

Moreover, SAC demonstrated no observable adverse effects in toxicological evaluations. In rodent studies, signs of toxicity were only reported at supraphysiological doses exceeding 500 mg/kg body weight. Subacute toxicity assays established a no-observed-adverse-effect level of approximately 250 mg/kg under controlled experimental conditions, indicating a wide margin of safety for SAC administration [63].

SAC safety is also highlighted in in vitro experimental results, which have consistently demonstrated the lack of cytotoxicity in normal cell models. In particular, dose- and time-response assays showed that SAC was not cytotoxic even at ~1 mM concentration in hepatocytes (HEPG2 [64] and BRL-3A [65]), retinal pigment epithelial cells (ARPE-19 [66]), bovine aortic endothelial cells (BAE-1; up to 10 mM) [67], and cultured human cardiomyocytes (tested up to 50 µM) [68]. These results indicate that SAC does not compromise the viability of non-transformed cells, even when administered at relatively high concentrations.

Conversely, in cancer cell models, SAC exhibits a selective cytotoxic effect that becomes evident mainly at higher doses. For example, apoptosis induction was reported in MCF-7 breast cancer cells at ~2.2 mM [69], while in glioma cell lines (RG2 and C6), SAC significantly reduced cell viability in a concentration-dependent manner, with ~25–50% loss of viability observed between 100–750 µM [70]. Similarly, cytotoxic effects have been described in lung [71], ovarian [72], bladder cancer cell lines [73], hepatocarcinoma [74] cell lines at concentrations above approximately 1 mM.

Overall, these findings suggest that SAC is non-toxic to normal cells under experimental conditions, while it can exert dose-dependent antiproliferative and pro-apoptotic activity in tumor cells, highlighting its potential as a selective anticancer agent.

It should be noted that thiol-containing compounds, including SAC and hydrogen sulfide donors, may potentially interfere with tetrazolium-based viability assays (e.g., MTT, MTS) through direct chemical reduction of the dyes, leading to possible artifacts [75]. Appropriate cell-free controls or complementary assays are therefore recommended to confirm cell viability results.

### 4.2. Antioxidant and Anti-Inflammatory Properties

Among the multiple bioactive properties of SAC, antioxidant and anti-inflammatory activities are considered central to its protective effects, particularly in the context of metabolic syndrome. In this section, we briefly outline these mechanisms, summarized into four principal activities, while directing the reader to a more focused and comprehensive review for further detail [5]. Briefly, SAC exerts antioxidant and anti-inflammatory effects through multiple complementary pathways. First, SAC functions as an endogenous donor of the gasotransmitter H_2_S, a key modulator of vascular oxidative stress by simultaneously scavenging ROS, preserving mitochondrial function, and attenuating cell senescence [76]. Moreover, SAC has an antioxidant activity that is linked to the presence of a free thiol group, which can donate a proton to reactive electrophilic species, thereby neutralizing ROS or reducing their reactivity. In addition, SAC can induce the expression of antioxidant enzymes via activation of the Nrf2 pathway; SAC further contributes to redox homeostasis by modulating NO synthesis. Finally, SAC exhibits metal-chelating properties, binding divalent metals such as Fe^2^⁺ and Fe^3^⁺ in a concentration-dependent manner. This ability is particularly relevant because excessive iron and copper can catalyze the formation of ROS, leading to oxidative stress and mitochondrial dysfunction. Collectively, these mechanisms support the multifaceted antioxidant and anti-inflammatory roles of SAC. In the following sections, we will frequently refer back to these pathways when discussing the beneficial effects of SAC on the cellular and organ dysfunctions that characterize MetS.

### 4.3. Beneficial Effects of S-Allyl Cysteine in Insulin Resistance, Diabetes, and Metabolic Dysfunction-Associated Steatotic Liver Disease (MASLD): In Vivo and In Vitro Evidence

Over the years, both in vivo and in vitro studies have provided substantial evidence supporting SAC’s role in regulating key metabolic parameters and modulating signaling pathways in experimental models simulating insulin-resistance and T2DM conditions.

Early studies in streptozotocin (STZ)-induced diabetic animal models demonstrated that SAC significantly reduced blood glucose levels and improved plasma and pancreatic antioxidant enzyme activity [77,78]. These findings were among the first to highlight that SAC treatment exerts a therapeutic protective feature in diabetes by decreasing oxidative stress.

Further in vivo research expanded these findings, showing that SAC could improve insulin homeostasis and regulate iron metabolism in diabetic animals [79]. In addition, SAC’s effects on the hepatic cytochrome P450 2E1 system were investigated, revealing a positive modulation of enzymes involved in glucose and lipid metabolism, which are often dysregulated in diabetic states [80].

Moreover, the study by Takemura et al. [81] investigated the effects of SAC on T2DM and MASLD in Otsuka Long-Evans Tokushima Fatty rats. SAC was administered for 13 weeks (0.45% in the diet), and the results showed significant improvements in several metabolic parameters (reduced hemoglobin A1c, blood glucose, triglycerides, and LDL cholesterol levels; normalized plasma insulin levels); moreover, SAC stimulates the mRNA and protein expression of peroxisome proliferator-activated receptors α and γ, and inhibited pyruvate dehydrogenase kinase 4 in the liver. It also normalized the expression of sterol regulatory element-binding protein 1c and forkhead box O1 proteins, which are involved in lipid and glucose metabolism regulation. Interestingly, the authors conclude that the effects observed in the liver were not attributed to the antioxidant property of SAC.

According to these results, Naidu et al. demonstrated in rats with STZ- and nicotinamide-induced diabetes that SAC exerted beneficial effects comparable to gliclazide. These effects were primarily attributed to inhibition of the polyol pathway through reduced aldose reductase and sorbitol dehydrogenase activities, which in turn improved glycemic control, increased glutathione levels, and protected pancreatic β-cells [82].

Moreover, studies using SAC-enriched black garlic juice have demonstrated a reduction in blood glucose and improvements in pancreatic function in STZ-induced insulin-deficient mice, confirming SAC’s potential in enhancing insulin secretion and pancreatic β-cell health [83].

Overall, in vivo data indicate that SAC counteracts hyperglycemia-induced metabolic alterations, mainly through antioxidant activity, with additional evidence suggesting possible redox-independent modulation of intracellular and metabolic pathways [81,82].

The in vitro effects of SAC, both in biochemical and cellular assays, have also been widely studied, revealing several key mechanisms through which SAC exerts its beneficial effects. Firstly, in vitro biochemical assays demonstrated that aged garlic extract and S-allyl cysteine inhibited metal-catalyzed protein fragmentation and Advanced Glycation End-products (AGE) formation, with S-allyl cysteine also reducing carboxymethyllysine, a non-crosslinked advanced glycation end product derived from oxidative processes [84].

Further studies also demonstrated that S-allyl cysteine and *N*-acetyl cysteine (NAC) inhibit protein glycation and oxidative modifications. Mechanistically, their protective action has been mainly attributed to the formation of adducts with reactive carbonyl species, which shield proteins from glycation [85].

As the accumulation of AGEs, formed from highly reactive carbonyl compounds under hyperglycemic conditions, represents a major pathological consequence of hyperglycemia and contributes to increased oxidative stress and inflammation [86], these experimental findings suggest that SAC may have potential for the treatment or management of metabolic syndrome.

In addition to directly counteracting hyperglycemia-associated damage, SAC has been shown to protect against hepatocyte dysfunctions related to MASLD. Specifically, SAC reduces hepatic lipid accumulation and lipotoxicity by activating AMPK and inhibiting SREBP-1-mediated lipogenesis [87].

Interestingly, a recent study shows that SAC reduced intestinal amyloid-beta synthesis in diabetic db/db mice, supporting its role in mitigating diabetes-enhanced amyloid-beta accumulation and Alzheimer’s disease progression [88].

In recent years, synergistic effects between SAC and other compounds have been explored. In particular, a very recent study investigated the combination of SAC with astaxanthin: the results highlight that Astaxanthin–S-allyl cysteine (AST-SAC) protects pancreatic β-cells from glucolipotoxicity by reducing oxidative stress, stabilizing mitochondria, and mitigating endoplasmic reticulum stress-mediated UPR signaling. AST-SAC preserves mTOR pathway function and insulin secretion, promoting β-cell survival under high glucose and palmitate exposure [89]. This combination further emphasizes SAC’s potential as part of multi-targeted therapeutic strategies for diabetes management.

Finally, our very recent findings highlight a novel and compelling mechanism through which SAC may exert its effects on insulin-dependent tissues. In cultured skeletal myotubes, SAC was found to enhance insulin signaling and glucose uptake, likely through a direct interaction with the catalytic site of the insulin receptor. Furthermore, in this cellular model, SAC effectively prevented and reversed palmitate-induced insulin resistance [90].

In conclusion, in vivo and in vitro studies (Table 1) consistently support the beneficial effects of S-allyl cysteine in the management of insulin resistance, diabetes, and related metabolic disorders. SAC’s ability to modulate oxidative stress, improve insulin secretion, protect pancreatic β-cells, regulate glucose and lipid metabolism and stimulate insulin-dependent pathway, underscores its potential as a multi-target therapeutic agent.

### 4.4. Beneficial Effects of S-Allyl Cysteine in Endothelial Dysfunction (ED)

Loss of endothelium physiological functions, as occurs in ED (see above, Section 3.3), prevents regular monitoring of vascular tone, also due to an increase in ROS and failure to release the gasotransmitters NO and H_2_S properly. Therefore, given the role of H_2_S in ED (see, for example, Citi et al. [91], Sun et al. [92]), as a potential natural source of H_2_S, SAC could play an important role in ED treatment, restoring H_2_S levels. However, while properties of SAC to counteract oxidative stress, inflammation, cancer, neurodegeneration, and cardiovascular diseases have been largely described, as we have already reported, its specific implication in ED has been the subject of a few studies (Table 2).

Taking into account the known mechanisms of its action, the protective effects of SAC against ED depend mainly on its antioxidant and anti-inflammatory potential. SAC has been demonstrated to suppress LDL oxidation, which causes damage to endothelial cells (ECs). When bovine pulmonary artery and human umbilical vein ECs were exposed to harmful treatment with oxidized LDL (Ox-LDL), the results of in vitro assays, concerning cell-membrane damage, mitochondrial injury, and lipid peroxidation, demonstrated that pretreatment of ECs with SAC minimized all negative effects after Ox-LDL treatment. Besides these anti-dyslipidemic actions, SAC restored glutathione (GSH) levels and reduced peroxide release, protecting cells from oxidant injury; finally, SAC inhibited expression of the pleiotropic nuclear transcription factor NF-kB, induced in HUVECs by TNF-α stimulation [93,94,95,96].

Activation of the NF-kB due to pro-inflammatory stimuli, such as in atherosclerotic lesions, could induce the expression of inducible NOS (iNOS) and NO production in macrophages and foam cells: noteworthy, it has been demonstrated that SAC is able to suppress NF-kB activation in LPS-activated macrophages [97]. In this work, Kim et al. also demonstrated that, in HUVECs, SAC induced an increase in NO production, assessed through evaluation of GMP levels, in a dose-dependent manner, while protein levels and cellular distribution of eNOS were not affected. Formation of hydroxyl radical and superoxide anion was also lowered by SAC [97]. Similar results about antioxidant and anti-inflammatory properties of SAC on macrophages and HUVEC were also obtained by Ho et al.: SAC, in a dose-dependent manner, inhibited oxidation of LDL, production of hydrogen peroxide, and activation of NF-kB [98]. These findings in both cell types suggested that SAC could have a protective effect in maintaining endothelial homeostasis and preventing the development of atherosclerotic plaques, thus protecting against ED.

In addition, as a capacity related to the prevention of ED as well as other consequent cardiovascular diseases, such as atherosclerosis and ischemia, SAC has been found to induce proliferation and tubulogenesis of progenitors of ECs in vitro with activation of AKT/eNOS signaling cascades, while in vivo, in mouse xenografts and ischemic models, SAC stimulated neovasculogenesis and restored endothelial functions in ischemic tissues [99].

Moreover, in another in vivo study, SAC has been shown to reduce ED in diabetic animals. In particular, in streptozotocin-nicotinamide-induced diabetic rats, SAC administration ameliorated all parameters related to diabetes, including restoration of the normal vascular structure as demonstrated by histological analysis of aorta rings [91].

The protective role exerted directly by H_2_S on HUVEC against cellular stress induced by hydrogen peroxide was reported by Wen et al.: ATP production, mitochondrial structure, ROS levels, and protein expression of antioxidant enzymes were preserved by H_2_S, which in this study was administered to ECs via its chemical donor NaSH [100].

In fact, there are few reports directly showing H_2_S intracellular release after treatment with SAC: for example, in a cellular model not related to ED, breast cancer cells, SAC displayed antiproliferative effects in a dose and time-dependent manner; moreover, SAC increased expression of enzymes involved in H_2_S release, in particular 3-mercaptopyruvate sulfurtransferase (MPST) [101].

In a previous work, on bovine aortic endothelial cells, we demonstrated that SAC significantly increases H_2_S release as evaluated in fluorescence microscopy experiments through a specific probe, SF7-AM. Following H_2_S synthesis, SAC reduced ROS levels produced by the oxidative stress-inducing menadione, and enhanced NO availability, by promoting eNOS phosphorylation and the consequent NO release, therefore emphasizing the role of SAC in maintaining endothelial homeostasis [67].

**Table 2:** Beneficial Effects of S-Allyl Cysteine in Endothelial Dysfunction.

Main Effect	Experimental Model	SAC Administration	References
Decrease in LDL oxidation levels Increase in GSH levels and decrease in peroxide release Inhibition of NFkB	Bovine pulmonary artery ECs and HUVECs	1–20 mM	[93,94,95,96]
Increase in NO production Decrease in hydroxyl radical and superoxide anion levels	HUVECs	20–80 µM	[97]
Inhibition of LDL oxidation, hydrogen peroxide production, and NFkB activation	HUVECs	0.1–10 mM	[98]
Activation of AKT/eNOS signaling cascades Stimulation of neovasculogenesis	Human endothelial progenitor cells (EPCs)/Neovasculogenesis xenograft model mice	10–150 µM/ 0.2 or 2 mg/kg	[99]
Increase in NO production Increase GSH, SOD, and CAT levels Restoration of the normal vascular structure in aorta rings	STZ and nicotinamide-induced diabetic male Wistar rats	150 mg/kg for 45 days	[102]
Increase in H_2_S production Decrease in ROS levels Increase in eNOS phosphorylation and NO production	Bovine aortic endothelial cells (BAE-1)	100 µM	[67]

### 4.5. Beneficial Effect of S-Allyl Cysteine in Gut Dysbiosis

Given the role of GM in the pathogenesis of MetS, garlic and, in particular, SAC, may represent a bioactive compound with a pivotal role in modulating GM composition. Escribano et al. demonstrated that SAC exerts a dose-dependent protective effect against oxidative damage, inflammation, and gut microbiota alterations in an experimental autoimmune encephalomyelitis (EAE) model. In particular, 50 mg/kg SAC treatment significantly reduced lipopolysaccharide (LPS) bacteria and LPS-binding protein (LBP) levels, both considered key biomarkers of gut dysbiosis [103]. Ried et al. investigated the effect of a daily intake of aged-garlic extract (1.2 g containing 1.2 mg SAC) on blood pressure, inflammation, and gut microbiota in 49 participants in a double-blind randomized placebo-controlled trial of 12 weeks. The results show that the garlic extract improved the microbial richness and diversity with a marked increase in *Lactobacillus* and *Clostridia* species after 3 months of supplementation [104]. Although current studies investigating the effect of SAC on GM are still limited and further research is needed, SAC may represent a potential modulator of GM, which is an increasingly recognized hallmark of MetS.

## 5. Conclusions

**Figure 2:**
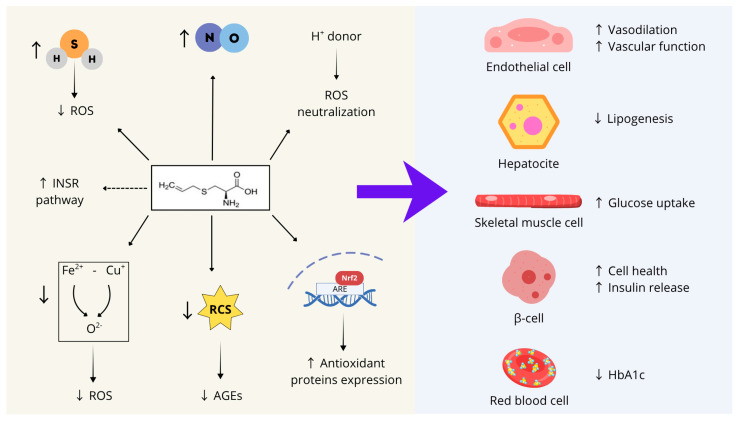
SAC’s biochemical features and main activities on different cell types whose dysfunction contributes to MetS development. ROS: reactive oxygen species; INRS: insulin receptor; RCS: reactive carbonyl species; AGEs: advanced glycation end-products; Nrf2: Nuclear factor erythroid 2-Related Factor 2; ARE: Adenylate-uridylate (AU)-rich element; ↑: Increase; ↓: Decrease. Figure created with Canva.com.

In conclusion, as summarized in Figure 2, S-allyl cysteine (SAC) can be proposed as a promising compound to support several physiological processes whose impairment contributes to the onset of MetS, including endothelial function, glucose and lipid metabolism, insulin responsiveness, and pancreatic β-cell health.

These characteristics stem from SAC’s biochemical features and can be summarized into seven main activities: (1) SAC acts as an endogenous hydrogen sulfide (H_2_S) donor, contributing to the cellular redox balance by scavenging ROS and preserving mitochondrial function; (2) SAC directly neutralizes ROS through its thiol group, which serves as a proton donor; (3) SAC activates the transcription factor Nrf2, thereby inducing the expression of several antioxidant enzymes; (4) SAC stimulates nitric oxide synthesis; (5) SAC mitigates iron- and copper-dependent oxidative stress; (6) SAC counteracts the formation of advanced glycation end-products (AGEs) by forming adducts with reactive carbonyl species; (7) SAC may directly interact with the catalytic site of the insulin receptor, enhancing its basal activity.

Moreover, recent studies have shown that SAC positively modulates gut microbiota composition, whose imbalance is commonly observed in patients with MetS.

Evidence from both preclinical and clinical studies supports these findings and underscores the multifaceted properties of SAC, which collectively counteract oxidative stress, inflammation, and insulin resistance.

Finally, SAC exhibits high bioavailability, remarkable plasma stability, and a wide safety margin in toxicological tests, further emphasizing its potential as a multi-target therapeutic agent for metabolic disorders.

## Figures and Tables

**Table 1:** Beneficial Effects of S-Allyl Cysteine in Insulin Resistance, Diabetes, and Metabolic Dysfunction-associated Steatotic Liver Disease: In Vivo and In Vitro Evidence.

Main Effect	Experimental Model	SAC Administration	References
Decrease in blood glucose and increase in plasma insulin levels Increase in plasma and pancreatic antioxidant enzyme activity Increase in plasma concentrations of ferritin, bilirubin, and iron, and a decrease in transferrin level Increase in leptin and adiponectin levels Increase in liver P450 2E1 activity	Streptozotocin (STZ)-induced diabetic adult Wistar strain albino male rats	150 mg/kg body weight for 45 days	[77,78,79,80]
Decrease in blood glucose, insulin, and hemoglobinA1c levels Decrease in LDL cholesterol and triglyceride levels Increase in mRNA and protein expression of PPARα and γ Increase in protein involved in lipid and glucose metabolism regulation	Otsuka Long-Evans Tokushima Fatty rats	0.45% dietary mixture for 13 weeks	[81]
Decrease in blood glucose and increase in plasma insulin levels Decrease in lipid peroxidation products and increase in the nonenzymatic antioxidant levels	STZ and nicotinamide-induced diabetic male Wistar rats	150 mg/kg body weight for 45 days	[82]
Decrease in blood glucose levels Decrease in visceral fat depots Increase in pancreatic insulin content and suppression of β-cell apoptosis	Streptozotocin (STZ)-induced diabetic male C57BL/6J mice	SAC-enriched black garlic juice (200 mg/kg) for 31 days	[83]
Inhibition of metal-catalyzed protein fragmentation and AGE formation Decrease in carboxymethyllysine levels	In vitro biochemical assays	0-84 mg/mL of aged garlic extract for 7/21/35 days	[84]
Inhibition of protein glycation and oxidative modifications	HT22 cells	0.25–1 mM	[85]
Inhibition of FFA-induced hepatocyte injury Decrease in FFA-induced lipid accumulation levels Decrease in protein expression levels of SREBP-1c and FAS	HepG2 cells	0.5–10 mM	[87]
Decrease in plasma amyloid-β levels	Male db/db and db/ + C57BLK/6 J mice	0.04% *w*/*w*	[88]
Decrease in oxidative stress levels Decrease in DNA fragmentation levels Increase in gene expression levels in correlation with insulin secretion	*Mus musculus* pancreatic β-cell line	5–15 μg/mL of AST-SAC	[89]
Increase in insulin signalling and glucose uptake Prevention and reversion of palmitate-induced insulin resistance	C2C12 cells	100 μM	[90]

## Data Availability

No new data were created or analyzed in this study. Data sharing is not applicable to this article.

## Abbreviations

The following abbreviations are used in this manuscript:

BMI	body mass index
CVDs	cardiovascular disease
EC	endothelial cell
ED	endothelial dysfunction
eNOS	endothelial nitric oxide synthase
FFA	free fatty acid
GM	gut microbiota
GSH	glutathione
H2S	hydrogen sulfide
IR	insulin resistance
LDL	Low-Density Lipoprotein
MASLD	Metabolic Dysfunction-Associated Steatotic Liver Disease
MedDiet	Mediterranean Diet
MetS	Metabolic Syndrome
NO	nitric oxide
ROS	reactive oxygen species
SAC	S-allyl cysteine
T2DM	type II diabetes
